# A retrospective analysis of the clinical profile and factors associated with mortality and poor hospital outcomes in adult Guillain–Barre syndrome patients

**DOI:** 10.1038/s41598-024-65265-0

**Published:** 2024-07-05

**Authors:** Zinabu Derso Tewedaj, Dawit Kebede Huluka, Yabets Tesfaye Kebede, Abel Tezera Abebe, Meksud Shemsu Hussen, Bekri Delil Mohammed, Leja Hamza Juhar

**Affiliations:** 1 Department of Internal Medicine, College of Health Science, Mekelle University, Mekelle, Ethiopia; 2 Division of Pulmonary and Critical Care Medicine, Department of Internal Medicine, College of Health Sciences, Addis Ababa University, Addis Ababa, Ethiopia; 3 Department of Medicine, Faculty of Medical Sciences, Institute of Health, Jimma University, Jimma, Ethiopia; 4 Department of Internal Medicine, Ethio-Tebib General Hospital, Addis Ababa, Ethiopia; 5 Department of Internal Medicine, St. Paul’s Hospital Millennium Medical College, Addis Ababa, Ethiopia

**Keywords:** Guillain–Barré syndrome, Ethiopia, Clinical profile, Outcomes, Prognostic factors, Diseases of the nervous system, Demyelinating diseases

## Abstract

Guillain–Barré syndrome (GBS) is an acute autoimmune polyneuropathy with substantial geographic variations in demography, antecedent events, clinical manifestations, electrophysiological sub-types, diagnostic findings, treatment modalities, and prognostic indicators. However, there is limited contemporary data on GBS patient profiles and prognostic factors from low-resource settings like Ethiopia. The objective of this study is to investigate the clinical profile, factors associated with mortality, and hospital outcomes among GBS patients admitted to Tikur Anbessa Specialized Hospital (TASH) in Addis Ababa, Ethiopia. A retrospective cross-sectional study was conducted among 60 GBS patients admitted to TASH from January 2018 to December 2022. Data on demographics, clinical features, treatments, complications, and outcomes were extracted from medical records. Bivariate and multivariate logistic regression analyses identified factors associated with mortality and poor hospital outcomes. The cohort had a mean age of 28.5 years, with 76.7% aged 14–34 years. Males comprised 61.7% of cases. Ascending paralysis (76.7%) was the predominant presentation. Absent or reduced reflexes were seen in 91.7% of patients. The most common antecedent event was gastroenteritis (26.7%), followed by upper respiratory tract infection (URTI) (15%) and vaccination (11.7%). The mean interval from symptom onset to hospital presentation was 8.77 days, and the peak symptom severity was 4.47 days. The axonal variant (75.5%) was the most common subtype, followed by the demyelinating variant (24.5%). Intravenous immunoglobulin was administered to 41.7% of patients. Respiratory failure requiring invasive mechanical ventilator (MV) support occurred in 26.7% of cases. The mortality rate was 10%, with mechanical ventilation being the only factor significantly associated with mortality (95% CI 2.067–184.858; P < 0.010). At discharge, 55% had a good outcome, and 45% had a poor outcome, according to the Hughes Functional Disability Scale (HFDS). Mechanical ventilation (AOR 0.024, 95% CI 0.001–0.607) and a GBS disability score > 3 (AOR 0.106, 95% CI 0.024–0.467) were factors significantly associated with poor hospital outcomes. GBS in this cohort primarily affected individuals of young age, commonly preceded by gastroenteritis and characterized by a high frequency of the axonal variant. Mechanical ventilation was found to be significantly linked to mortality. Alongside mechanical ventilation requirements, severe disability upon presentation emerged as a crucial determinant of poor outcomes upon discharge, underscoring the importance of early identification of high-risk patients and prompt interventions.

## Introduction

Guillain–Barré syndrome (GBS) is an acute polyradiculoneuropathy characterized by immune-mediated damage to the peripheral nervous system, leading to varying degrees of motor dysfunction, sensory impairment, and autonomic instability ^1^. It represents the most common cause of acute flaccid paralysis globally, exerting a substantial burden on healthcare systems due to the intensity of care required during the acute phase and the long-term rehabilitation requirements ^2,3^. Epidemiological data from North America and Europe indicate an annual incidence of GBS ranging from 0.8 to 1.9 cases per 100,000 person-years ^4^.

GBS exhibits notable variations in incidence, demographic distribution, preceding events, clinical manifestations, electrophysiological subtypes, diagnostic approaches, therapeutic interventions, and prognostic outcomes across different geographical regions ^5–8^. These variations can be attributed to multifaceted factors. Firstly, regional differences in the prevalence and strains of infectious agents such as cytomegalovirus (CMV), Epstein-Barr virus (EBV), and Campylobacter contribute to regional discrepancies in GBS incidence rates ^9^. Moreover, variations in hygiene practices across regions affect exposure to these pathogens, potentially influencing GBS development ^10^. Dietary habits and nutrient deficiencies also affect disease progression ^11^. Environmental factors unique to specific regions also serve as potential triggers for the onset of GBS ^12^. Furthermore, genetic variations among populations influence susceptibility to GBS and disease severity ^12,13^. In regions with limited access to advanced diagnostic tools, underdiagnosis or misdiagnosis of GBS subtypes may occur, impacting reported incidence rates ^10^. Moreover, slight differences in diagnostic criteria and disease reporting practices across regions further complicate the accurate assessment of GBS burden ^5,10^. The absence of affordable and effective treatments significantly worsens outcomes in low- and middle-income countries. Furthermore, socioeconomic factors such as poverty, inadequate infrastructure, and healthcare disparities further compound the difficulties in accessing timely and appropriate care ^10^.

Most comprehensive studies investigating GBS patient profiles and outcomes originate from high-income regions, particularly North America and Europe. Consequently, there exists a need for more contemporary data on GBS from low- and middle-income countries, including Africa, with limited representation from Ethiopia, thereby impeding a comprehensive understanding of geographical variations in the disease. Moreover, existing studies from the region need to be updated, to accurately depict the current GBS landscape in Ethiopia.

This study aims to address this gap in the literature by thoroughly investigating the clinical profile and factors associated with mortality and hospital outcomes among patients diagnosed with GBS admitted to Tikur Anbessa Specialized Hospital (TASH), Ethiopia. By elucidating the contemporary epidemiological, clinical, and prognostic features of GBS in the Ethiopian context, this research endeavors to provide invaluable insights into managing and treating the condition within the local healthcare setting.

## Methodology

### Study design and setting

A retrospective cross-sectional chart review study was conducted at TASH, focusing on patients admitted to the medical intensive care unit (MICU) and medical ward who were diagnosed with GBS during the period from January 1, 2018, to December 30, 2022. The inclusion criteria encompassed patients aged 14 years and older whose clinical records provided comprehensive information. Excluded from the study were individuals with missing and incomplete medical documentation. Data encompassing clinical and paraclinical variables, inclusive of sociodemographic factors, primary presenting symptoms, symptom and in-hospital stay duration, antecedent events, complications, utilized treatment modalities, mechanical ventilation requirement, and investigation outcomes such as lumbar puncture cytochemistry and nerve conduction studies, were obtained.

Patients were stratified based on GBS diagnostic certainty as per Brighton’s criteria ^14^, alongside their functional status at hospital admission, assessed utilizing the Hughes Functional Disability Scale (HFDS), also known as the GBS disability score ^15,16^ (see Supplementary Table S1). The classification of patients' nerve conduction studies into electrophysiological variants of GBS relied on Rajabally's electrophysiological criteria following a single nerve conduction study ^17^.

### Operational definitions

In our study, dysautonomia is defined by the presence of blood pressure fluctuations (hypertension or hypotension), occurrences of postural hypotension (a drop of 20 mmHg in systolic blood pressure or 10 mmHg in diastolic blood pressure within 5 min of rising from a supine or seated position), and manifestations of cardiac dysrhythmias (tachycardia or bradycardia) attributable solely to autonomic nervous system dysfunction ^18,19^. Assessment of the need for mechanical ventilator support encompassed evaluations of respiratory rate, single breath count, incapacity to lift the head, and oxygen saturation levels. A poor outcome was identified by the inability to ambulate independently, denoted by a GBS disability score of 3 or higher upon hospital discharge ^20^.

### Ethical approval

The present research received ethical clearance from the Institution of Health Research Ethics Review Committee of Tikur Anbessa Specialized Hospital, Internal Medicine Department. The study was conducted in strict accordance with the relevant guidelines and regulations set forth by the committee. Informed consent was waived by the Institutional Health Research Ethics Review Committee of TASH due to the retrospective nature of the study, following established protocols.

### Statistical analysis

We utilized SPSS version 26 for data analysis. Before analysis, data completeness was ensured. Socio-demographic characteristics were presented in tabular format, detailing both numbers and percentages. A bivariate analysis was conducted to identify independent variables at a significance level of 5%, which were subsequently incorporated into the multivariate binary logistic regression analysis. In the multivariate logistic regression, a 95% confidence interval was calculated for the adjusted odds ratio (AOR), with variables exhibiting a p-value ≤ 0.05 considered statistically associated with poor hospital outcomes among GBS patients.

### Ethics approval and consent to participate

Ethical clearance for the study was obtained from the Institution of Health Research Ethics Review Committee of Tikur Anbessa Specialized Hospital, Internal Medicine Department. Officials at various levels within the study area were duly informed through official letters issued by the Internal Medicine Department. Throughout the study, strict measures were implemented to uphold the confidentiality of collected information, and the privacy of participants was meticulously maintained, ensuring compliance with ethical standards and safeguarding the rights of all involved individuals. Informed consent was waived due to the retrospective nature of the study by the Institutional Health Research Ethics Review Committee of TASH.

## Results

**Figure 1:**
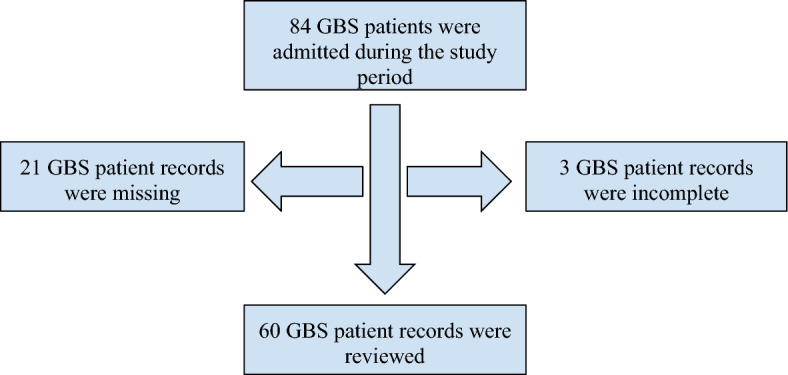
Flow chart showing the number of identified and excluded medical records of patients.

During the study period spanning from January 2018 to December 2022, a total of 60 GBS patient charts were thoroughly reviewed and included in the analysis for the study (see Fig. 1).

### Sociodemographic and clinical profile of patients

The study exhibited a mean age of 28.5 ± 12.5 years, ranging from 14 to 70 years. The male-to-female ratio was calculated as 1.61, with males comprising 37 individuals (61.7%). Analysis of the age distribution revealed that most cases, comprising 46 (76.7%), fell within the age bracket of 14–34 years (see Table 1).

**Table 1:** Socio-demographic and clinical profile of GBS patients in TASH, Addis Ababa, Ethiopia, Jan 2018 – Dec 2022 (n = 60).

Variables		Frequency n (%)
Age group	14–34	46 (76.7%)
	35–55	11 (18.3%)
	> 55	3 (5%)
Presenting symptoms	Ascending weakness	46 (76.7%)
	Descending weakness	14 (23.3%)
	Dysphagia	11 (18.3%)
	Sensory involvement	10 (16.7%)
	Respiratory failure	9 (15%)
	Bladder involvement	8 (13.3%)
	Facial nerve palsy	6 (10%)
Deep tendon reflexes	Absent/hyporeflexia	55 (91.7%)
	Normal	5 (8.3%)
	Hyperreflexia	0 (0%)
Antecedent events	None	27 (45%)
	Gastroenteritis	16 (26.7%)
	URTI	9 (15%)
	Vaccination	7 (11.7%)
	COVID-19	1 (1.7%)

Ascending weakness emerged as the predominant presenting symptom among GBS patients, accounting for 46 (76.7%) cases. Bulbar nerve involvement (cranial nerves IX and X) resulting in dysphagia was observed in 11 patients (18.3%), while cranial nerve VII involvement causing facial palsy was noted in 6 patients (10%). Details are provided in Table 1.

The primary antecedent event identified in this study was gastroenteritis, observed in 16 (26.7%) cases. Post-vaccination GBS was seen in 7 (11.7%) cases. Of the 7 vaccination instances, 6 pertained to anti-rabies vaccines and 1 to the COVID-19 vaccine. Notably, COVID-19 infection preceded the onset of GBS in 1 patient. Conversely, 27 (45%) patients exhibited no antecedent infection. 16 (26.7%) patients required mechanical ventilation (see Table 1). Additionally, six patients presented with comorbid illnesses, including 4 cases of hypertension (HTN), 1 case of dilated cardiomyopathy (DCMP), and 1 case of chronic myeloid leukemia (CML).

The mean interval from the onset of symptoms to presentation at the hospital was 8.77 (± 7.25) days, ranging from 1 to 40 days. Additionally, the mean duration from the initial symptom to peak symptomatology was 4.47 (± 4.78) days, ranging from 1 to 21 days. Hospitalization durations varied widely, ranging from 2 to 180 days, with a mean stay of 26.08 (± 31.08) days. Among the 16 patients who required mechanical ventilation (MV) support, the mean duration of MV support was 25.50 (± 18.79) days, ranging from 8 to 82 days.

### Diagnosis, laboratory, and nerve conduction profile of patients

Regarding the laboratory tests, lumbar puncture was conducted on 47 patients, revealing albuminocytological dissociation in 39 cases (82.9%). Nerve conduction studies were performed on 45 individuals. The predominant GBS variant observed in this study was the axonal variant, present in 34 out of 45 cases (75.5%), followed by the demyelinating variant in 11 out of 45 cases (24.5%). Among the axonal variant cases, 28 cases (82.3%) were classified as acute motor axonal neuropathy (AMAN), while 6 cases (17.7%) were classified as acute motor and sensory axonal neuropathy (AMSAN). None of the 33 patients who underwent serological testing for HIV yielded reactive results (see Table 2).

**Table 2:** Lumbar puncture and nerve conduction study of GBS patients in TASH, Addis Ababa, Ethiopia, Jan 2018 – Dec 2022 (n = 60).

Variables		Frequency n (%)
Lumbar puncture with albuminocytological dissociation	Yes	39 (65%)
	Not done	13 (21.7%)
	No	8 (13.3%)
Nerve conduction study variant of GBS	Axonal	34 (56.7%)
	Not done	15 (25%)
	Demyelinating	11 (18.3%)
HIV test	Non-reactive	33 (55%)
	Not done	27 (45%)
	Reactive	0

**Figure 2:**
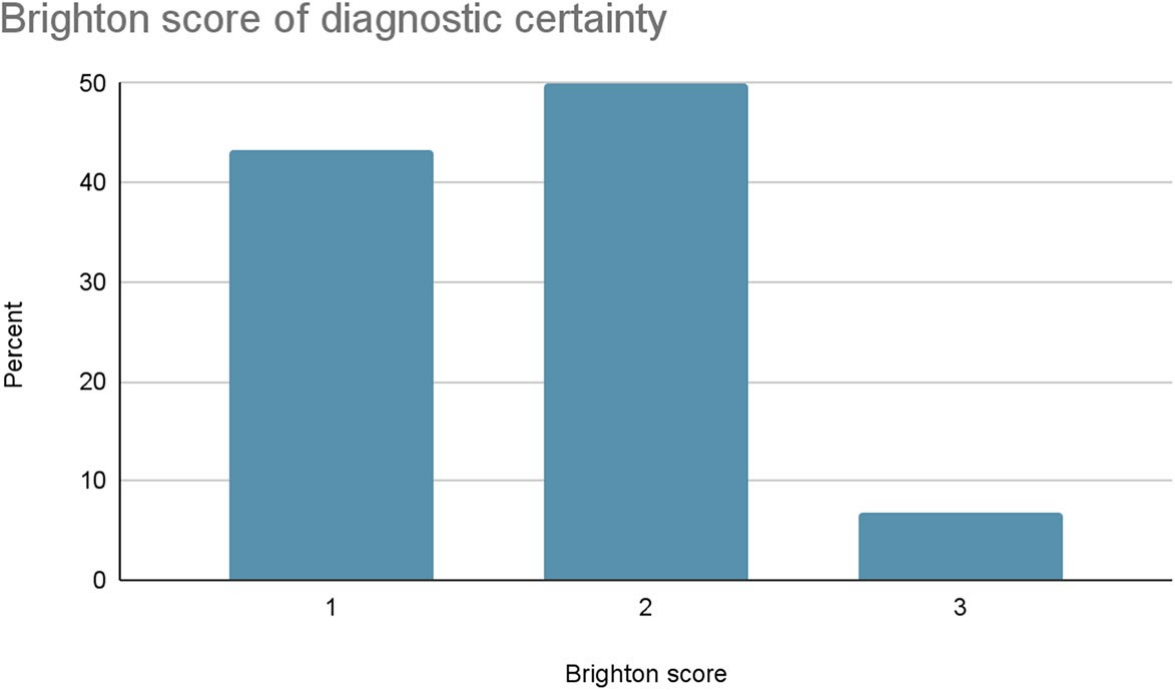
Brighton criteria level of diagnostic certainty of diagnosis of GBS in TASH, Addis Ababa, Ethiopia, Jan 2018–Dec 2022 (n = 60).

The diagnostic certainty of patients in this study is depicted in Fig. 2. A Brighton score of 2 was the most common score, observed in half of the patients, totaling 30 cases (50%). Similarly, nearly half of the patients had a Brighton score of 1, comprising 26 cases (43.3%).

### Treatment and outcomes of patients

Intravenous immunoglobulin (IVIg) treatment was administered to 25 patients, accounting for 41.7% of the cohort. Additionally, one patient received steroids for a severe hospital-acquired infection, while none of the patients underwent plasmapheresis. Notably, specific treatment was not provided to 34 patients (56.7%), with only supportive care being administered.

Upon bivariate logistic regression analysis, IVIg treatment did not demonstrate an association with either death (p = 0.22) or hospital outcome (p = 0.90). Furthermore, a Mann–Whitney U test revealed that the length of hospital stays for patients receiving IVIg (mean rank = 34.9 days) was not significantly different from those not receiving IVIg (mean rank = 27.3 days), with a p-value of 0.096).

Despite a shorter duration of mechanical ventilation support observed in patients who received IVIg (mean = 20.6 days, SD = 11.7 days) compared to those who did not (mean = 30.3 days, SD = 23.7 days), this difference was not statistically significant according to t-test analysis (t(16) = − 1.041, p = 0.316).

The hospital mortality rate among patients diagnosed with GBS in this study was determined to be 10%, with 6 out of 60 patients succumbing to their condition. The causes of death were attributed to sudden cardiac arrest in 3 patients, respiratory arrest in 2 patients, and uncontrolled urosepsis in 1 patient. Notably, the requirement for mechanical ventilation support was significantly associated with death on bivariate analysis (5 out of 6 cases; 95% CI 2.067–184.858; p < 0.010).

Common complications observed in this study included infections in 19 cases (31.7%), comprising catheter-associated urinary tract infections (CA-UTI) in 12 cases, hospital-acquired pneumonia (HAP) in 10 cases, COVID-19 infection in 1 case, and thrombophlebitis in 1 case. Autonomic dysfunction was noted in 17 cases (28.3%), while bed sores were observed in 4 cases (6.7%). Additionally, tracheoesophageal fistula (TEF) occurred in 3 cases (5%), and pneumothorax was documented in 2 cases (3.3%).

Among the total of 60 patients admitted in this study, 33 patients (55%) had a good outcome at discharge, while 27 patients (45%) experienced a poor outcome, as indicated by a high Hughes score.

### Factors associated with poor hospital outcome (high Hughes score) at discharge

In bivariate binary logistic regression analysis conducted at a 95% level of significance (p < 0.05), several factors were identified as significantly associated with poor hospital outcomes. These factors included respiratory failure at presentation, the requirement for MV support, autonomic dysfunction, infection, and a GBS functional disability score > 3 at admission, as delineated in Table 3.

**Table 3:** Bi-variate and multi-variate analysis for associated factors with poor hospital outcome of GBS patients in TASH, Addis Ababa, Ethiopia, Jan 2018–Dec 2022 (n = 60).

Variables		Hughes scale at discharge		COR (95% CI)	AOR (95% CI)	p-value
		Good outcome	Bad outcome			
Respiratory failure at presentation	No	31 (60.7%)	20 (39.3%)	1	1	
	Yes	2 (22.3%)	7 (77.7%)	0.184 (0.035–0.978)	1.747 (0.107–28.619)	0.696
Requiring MV support	No	32 (72.7%)	12 (27.3%)	1	1	
	Yes	1 (6.25%)	15 (93.75%)	0.025 (0.003–0.210)	0.024 (0.001–0.607)	0.024*
Autonomic dysfunction	No	28 (65.1%)	15 (34.9%)	1	1	
	Yes	5 (29.4%)	12 (70.6%)	0.223 (0.066–0.754)	0.772 (0.103–5.756)	0.801
Infection	No	28 (68.3%)	13 (31.7%)	1	1	
	Yes	5 (26.3%)	14 (73.7%)	0.166 (0.049–0.559)	0.997 (0.129–7.715)	0.998
Disability at admission	HFDS ≤ 3	24 (80%)	6 (20%)	1	1	
	HFDS > 3	9 (30%)	21 (70%)	0.107 (0.033 – 0.351)	0.106 (0.024—0.467)	0.003*

*p-value < 0.05 on multivariate analysis, *HFDS* Hughes functional disability scale.

However, upon conducting multivariable binary logistic regression analysis, only the need for MV support and a GBS functional disability score > 3 at admission were found to be significantly associated with a poor hospital outcome at discharge (p < 0.05).

### Limitations

The limitation of our study is its retrospective nature, relying on chart reviews, which are contingent upon the accuracy and completeness of documentation. Additionally, the relatively small sample size represents another limitation, diminishing the statistical power of the findings and impeding their generalizability to broader patient populations.

## Discussion

### Sociodemographic and clinical profiles of patients

GBS affects all age groups, with prevalence generally increasing with age ^21,22^. While common in children, it is less frequent than in adults ^23^. Notably, studies show a bimodal distribution of the disease ^22,24^. The first peak occurs between ages 15 and 34, a trend corroborated by our study. The second peak occurs after age 50. Some studies reported mean ages of 30 and 29.3 years ^25,26^. Conversely, others have documented comparatively older mean ages, ranging from 40.69 to 52.6 years ^22,27–30^. The age-related variations in GBS may stem from immune system changes ^31^, declining nerve repair mechanisms ^32^, and varied exposure to infectious agents ^9^.

GBS is more prevalent in males than females, with ratios ranging from 1.1:1 to 1.7:1 ^23,33^. Interestingly, while girls and adolescent females are more likely to develop GBS, this trend reverses in older age groups ^34^. The higher prevalence in males may be due to sex differences in immune response, but factors like sex hormones, genetics, and environmental influences also play significant roles, warranting further investigation ^33^.

In our study, the predominant GBS presentation was ascending paralysis, consistent with other studies ^35–37^. The mean interval from symptom onset to hospital presentation in Ethiopia improved from 11.2 days two decades ago to 8.77 days in our study, likely due to better awareness and healthcare access ^38,39^. IVIg use increased to 41.7% from 6.2%, indicating improved treatment ^38^. However, the mean hospital stay remains longer than in Thailand (14.2 days) and the Netherlands (17 days), reflecting ongoing healthcare challenges in Ethiopia ^29,40^.

Albuminocytological dissociation (ACD), a hallmark diagnostic feature of GBS with reported incidences ranging from 44 to 81%, was observed in 82.9% of participants in our study ^14,41,42^. This high prevalence may be due to delayed healthcare presentation, lumbar puncture procedures conducted later in disease progression, and the absence of localized variants in our cohort ^43,44^.

In our study, the predominant variant of GBS was axonal, accounting for 75.5%. This aligns with findings from studies in northern China, India, and Mexico ^26,45,46^. However, it contrasts with studies in southern China, the Balkans, Wuhan-China, Thailand, and Canada, where acute inflammatory demyelinating polyneuropathy (AIDP) is more common. ^14,29,47,48^. The difference may be attributed to a higher prevalence of preceding gastroenteritis and a younger age distribution in our cohort, factors often associated with axonal variants.

### Factors associated with mortality and poor hospital outcomes

In our study, the observed mortality rate of 10% in GBS patients aligns with the reported range (1–18%) and is higher among those requiring mechanical ventilation (12–20%) ^49^. Mortality was primarily associated with the need for MV, reflecting the severity of nerve involvement and risks such as ventilator-associated pneumonia (VAP) and ventilator-induced lung injury (VILI) ^50,51^. These complications underscore the challenges and increased mortality risks associated with MV in GBS. Additionally, a significant subset (45%) experienced poor outcomes at discharge, characterized by a GBS disability score > 3 at discharge. Factors significantly associated with a poor hospital outcome (p < 0.05) include the requirement for MV support and a GBS disability score > 3 at admission. A GBS disability score > 3 at admission can exacerbate complications like pneumonia and deep vein thrombosis (DVT) ^52,53^. Early mobilization and proactive management strategies are crucial to mitigate these risks and improve patient recovery and outcomes.

## Conclusion

In conclusion, this retrospective cross-sectional study provides valuable insights into the contemporary clinical profile and factors influencing the outcomes and mortality of GBS patients in Ethiopia. The study addresses a notable gap in the literature by examining this neurological condition within the context of a low-resource setting. Key findings revealed a predominance of the axonal variant of GBS, with the majority of patients presenting with ascending paralysis. Mechanical ventilation requirements and a GBS disability score > 3 at admission emerged as significant risk factors associated with poor hospital outcomes. Moreover, the need for mechanical ventilation was identified as a predictor of mortality risk. While the observed overall mortality rate aligned with global estimates, a substantial proportion of discharged patients exhibited residual functional disability. These findings underscore the complexities of managing GBS and highlight the need for early identification of high-risk patients, prompt initiation of appropriate treatments, and the implementation of comprehensive rehabilitation strategies tailored to the local healthcare environment. By elucidating the challenges and prognostic factors in the Ethiopian context, this study provides a foundation for developing targeted interventions and optimizing resource allocation to improve care delivery and mitigate the burden of GBS in similar resource-constrained settings.

### Supplementary Information

Supplementary Table S1.

## Data Availability

The data supporting the findings of this study will be available from the corresponding author upon reasonable request.
