# Supplementary material for: A retrospective analysis of the clinical profile and factors associated with mortality and poor hospital outcomes in adult Guillain–Barre syndrome patients
Source: Sci Rep. 2024 Jul 5;14:15520. doi: 10.1038/s41598-024-65265-0 (PMC11226644; doi:10.1038/s41598-024-65265-0)
Supplement: Supplementary file 1 — Supplementary Table S1. [file 41598_2024_65265_MOESM1_ESM.docx]

**Table S1:** Levels of Brighton’s diagnostic certainty and scores of GBS functional disability according to Hughes functional disability scale

|  | **Classification** | **Interpretation** |
| --- | --- | --- |
| GBS diagnostic certainty per Brighton’s criteria | Level-1 | High diagnostic certainty |
|  | Level-2 | Moderate diagnostic certainty |
|  | Level-3 | Lower diagnostic certainty |
|  | Level-4 | Lowest diagnostic certainty |
| GBS functional disability per Hughes Functional Disability Scale | Score-0 | A healthy state |
|  | Score-1 | Minor symptoms and capable of running |
|  | Score-2 | Able to walk 10 meters or more without assistance but unable to run |
|  | Score-3 | Able to walk 10 meters across an open space with help |
|  | Score-4 | Bedridden or chairbound |
|  | Score-5 | Requiring assisted ventilation for at least part of the day |
|  | Score-6 | Dead |
